# Macrophage Depletion in Elderly Mice Improves Response to Tumor Immunotherapy, Increases Anti-tumor T Cell Activity and Reduces Treatment-Induced Cachexia

**DOI:** 10.3389/fgene.2018.00526

**Published:** 2018-11-06

**Authors:** Lelinh Duong, Hannah G. Radley-Crabb, Joanne K. Gardner, Federica Tomay, Danielle E. Dye, Miranda D. Grounds, Fiona J. Pixley, Delia J. Nelson, Connie Jackaman

**Affiliations:** ^1^School of Pharmacy and Biomedical Sciences, Faculty of Health Sciences, Curtin Health Innovation Research Institute, Curtin University, Perth, WA, Australia; ^2^School of Human Sciences, The University of Western Australia, Perth, WA, Australia; ^3^School of Biomedical Sciences, the University of Western Australia, Perth, WA, Australia

**Keywords:** macrophage, aging, cancer, immunotherapy, T cells, cachexia

## Abstract

Most cancers emerge in the elderly, including lung cancer and mesothelioma, yet the elderly remain an underrepresented population in pre-clinical cancer studies and clinical trials. The immune system plays a critical role in the effectiveness of many anti-cancer therapies in young hosts via tumor-specific T cells. However, immunosuppressive macrophages can constitute up to 50% of the tumor burden and impair anti-tumor T cell activity. Altered macrophage phenotype and function during aging may further impact anti-tumor T cell responses. Yet, the impact of macrophages on anti-tumor T cell responses and immunotherapy in the elderly is unknown. Therefore, we examined macrophages and their interaction with T cells in young (3 months) and elderly (20–24 months) AE17 mesothelioma-bearing female C57BL/6J mice during tumor growth. Mesothelioma tumors grew faster in elderly compared with young mice, and this corresponded with an increase in tumor-associated macrophages. During healthy aging, macrophages increase in bone marrow and spleens suggesting that these sites have an increased potential to supply cancer-promoting macrophages. Interestingly, in tumor-bearing mice, bone marrow macrophages increased proliferation whilst splenic macrophages had reduced proliferation in elderly compared with young mice, and macrophage depletion using the F4/80 antibody slowed tumor growth in young and elderly mice. We also examined responses to treatment with intra-tumoral IL-2/anti-CD40 antibody immunotherapy and found it was less effective in elderly (38% tumor regression) compared to young mice (90% regression). Tumor-bearing elderly mice decreased *in vivo* anti-tumor cytotoxic T cell activity in tumor draining lymph nodes and spleens. Depletion of macrophages using F4/80 antibody in elderly, but not young mice, improved IL-2/anti-CD40 immunotherapy up to 78% tumor regression. Macrophage depletion also increased *in vivo* anti-tumor T cell activity in elderly, but not young mice. All the tumor-bearing elderly (but not young) mice had decreased body weight (i.e., exhibited cachexia), which was greatly exacerbated by immunotherapy; whereas macrophage depletion prevented this immunotherapy-induced cachexia. These studies strongly indicate that age-related changes in macrophages play a key role in driving cancer cachexia in the elderly, particularly during immunotherapy, and sabotage elderly anti-tumor immune responses.

## Introduction

Life expectancy of the human global population is increasing. However, health deterioration with aging is associated with an increased incidence of cancers, including mesothelioma, in the elderly. The immune system plays a crucial role in eliminating cancerous cells, however, immune function can decline with age, termed “immunosenescence” (Pawelec and Solana, 1997). One thought is that immunosenescence occurs as a consequence of life-long exposure to antigenic stress and inflammation (Pawelec, 2012). Chronic, low grade inflammation is also reported with aging, referred to as “inflammaging” (Franceschi et al., 2007). This is characterized by increased circulating levels of pro-inflammatory cytokines, such as tumor necrosis factor (TNF)-α and interleukin (IL)-6 (Franceschi et al., 2000, 2007; Castelo-Branco and Soveral, 2014). Concurrently, elevated levels of anti-inflammatory cytokines, such as transforming growth factor (TGF)-β and IL-10, have been reported (Carrieri et al., 2004; Lio et al., 2004; Franceschi et al., 2007; Castelo-Branco and Soveral, 2014), which may regulate the inflammatory aging microenvironment. Disturbance of the balance between pro- and anti-inflammatory factors may impact on physiological function of the host and account for the increasing cancer rates in the elderly population. Furthermore, age-related changes in thymic function and T cells have also been proposed as a major contributor to the increased cancer incidence in older humans (Palmer et al., 2018).

The immune system plays a crucial role in host defense against cancer through recognition and elimination of cancerous cells primarily by tumor-specific cytotoxic T lymphocytes (CTLs; Chen and Mellman, 2013). Anti-tumor CTLs are generated by interaction with antigen presenting cells (APCs), including dendritic cells and macrophages (Chen and Mellman, 2013). Tumor antigen presentation by APCs and subsequent activation of T cells generally occurs in draining lymph nodes (DLNs) and effective tumor cell killing requires activated CTLs to traffic to the tumor site (Gardner and Ruffell, 2016). However, this complex process can fail due to the tumor employing escape mechanisms (Speiser et al., 2016), leading to disease progression. As a result, immunotherapies have been designed that aim to induce or enhance specific anti-tumor immunity mediated by CTLs (Rosenberg, 2014). Yet, T cell function is also compromised during aging (Koch et al., 2008; Fulop et al., 2010) and therefore some T cell-based immunotherapies in the elderly may be less effective.

Anti-tumor T cells must overcome an immunosuppressive tumor microenvironment in order to eliminate tumor cells (Speiser et al., 2016). Key mediators of immunosuppression in the tumor are tumor-associated macrophages (TAMs), which can make up to 50% tumor burden and are often correlated to poor prognosis (Luo et al., 2006; Zhang et al., 2012, 2016; Fritz et al., 2014). It has been proposed that tumor evolution starts with chronic inflammation wherein pro-inflammatory macrophages secreting cytokines such as IL-6 and TNF-α promote neoplastic transformation (Sica et al., 2008; Biswas and Mantovani, 2010). Tumor-derived factors such as C-C ligand 2 (CCL2) and colony stimulating factor-1 (CSF-1) can also recruit further macrophages to the tumor from the bone marrow (BM) and spleen (Cortez-Retamozo et al., 2012). Once established, tumor-derived factors drive macrophages toward an immunosuppressive phenotype which impairs anti-tumor T cell activity, primarily through the secretion of anti-inflammatory cytokines/chemokines such as TGF-β, IL-10, CCL17, and CCL22 (Biswas and Mantovani, 2010; Jackaman et al., 2017).

Several lines of evidence indicate dysregulated macrophage function with aging (Oishi and Manabe, 2016). For example, *in vitro* splenic macrophages from elderly female Balb/c mice (aged 18–20 months) were hyposensitive to pro-inflammatory lipopolysaccharide (LPS) stimuli, with reduced production of TNF-α and IL-1β compared to young mice (Mahbub et al., 2012). However, in separate studies BM-derived macrophages from elderly female C57BL/6J mice (aged 16–22 months) exhibited increased TNF-α and IL-6 production in response to LPS (Bouchlaka et al., 2013; Thevaranjan et al., 2017). Similarly, elderly derived female Balb/c (aged 17–18 months) and C57BL/6J peritoneal macrophages (aged 24–28 months) displayed hypersensitivity to anti-inflammatory IL-4 stimuli with increased production of anti-inflammatory IL-10 and TGF-β (Jackaman et al., 2013, 2014). *In vivo* murine studies have also described macrophages from the eye, muscles, lymph nodes, spleen and bone marrow expressing increased IL-10 during aging (Wang et al., 1995, 2015; Kelly et al., 2007; Jackaman et al., 2013). However, liver and adipose tissue macrophages from elderly male and female C57BL/6J mice (18–22 months) express increased pro-inflammatory IL-6 and TNF-α (Lumeng et al., 2011; Fontana et al., 2013). It is likely that each tissue microenvironment will exert distinct influences on macrophage phenotype during aging which may account for the discrepancies between these studies. However, the impact of the tumor microenvironment on macrophages during aging remains largely unknown.

We have previously shown that local intra-tumoral administration of IL-2/anti-CD40 immunotherapy induces mesothelioma tumor regression in young (aged 2–3 months) female C57BL/6J mice (Jackaman et al., 2008, 2016; Jackaman and Nelson, 2012); mediated primarily by neutrophils, CTLs and macrophages. This response included the generation of anti-tumor CTLs and polarization of macrophages into a pro-inflammatory phenotype within DLNs in young mice *in vivo* (Jackaman et al., 2016). Similarly, we have shown that IL-2/anti-CD40-activated macrophages can rescue age- and tumor-induced T cell function *in vitro* (Jackaman et al., 2013, 2014). However, it is likely that the inflammaging microenvironment plays a pivotal role in age-related macrophage dysfunction (Oishi and Manabe, 2016) and further *in vivo* studies are required.

Aging is a risk-factor for cancer-associated cachexia (Ali and Garcia, 2014; Wallengren et al., 2015), which is characterized by loss of body weight or skeletal muscle atrophy (Fearon et al., 2011). The diagnosis of cachexia often marks a decline in survival with poor responses to anti-cancer therapy and the severity is not necessarily associated to tumor size (Ross et al., 2004; Fearon, 2008, Fearon et al., 2012). Cancer cachexia is possibly due to metabolic dysfunction and is associated with the same inflammatory cytokines that are elevated with inflammaging (e.g., IL-6 and TNF-α; (Franceschi et al., 2000, 2007; Castelo-Branco and Soveral, 2014). Moreover, tumor- and host-derived cytokines, potentially released by TAMs, may contribute to cachexia (Billingsley et al., 1996; Fearon et al., 2012). However, few pre-clinical models have examined the contribution of macrophages to cancer cachexia in elderly mice.

Therefore, to address this issue we examined the role of macrophages in young (3 months) vs. elderly (20–24 months) mesothelioma-bearing female C57BL/6J mice during tumor growth and following treatment with intra-tumoral IL-2/anti-CD40 immunotherapy. We used flow cytometry to assess the percentage of macrophages in spleen, bone marrow (BM), and tumors, as well as macrophage proliferation via Ki67. Macrophages were depleted *in vivo* using F4/80 antibody and mice monitored for response to IL-2/anti-CD40 immunotherapy, as well as weight loss (i.e., cachexia). In order to assess the impact of macrophage depletion on T cell function, *in vivo* anti-tumor CTL analyses was performed in IL-2/anti-CD40 treated mice ± macrophage depletion. Taken together, our findings suggest that macrophages in the elderly facilitate faster mesothelioma tumor growth, leading to impairment of anti-tumor immune responses and cancer cachexia.

## Materials and Methods

### Mice

Female C57BL/6J mice aged 3 months (young, equivalent to 18-year-old humans) and 20–24 months (elderly, equivalent to 60–70-year-old humans), as defined by the Jackson Laboratory (Yuan et al., 2009), were obtained from the Animal Resources Center (ARC, Murdoch, WA, Australia). In this study we analyzed 20–24 months as the lifespan of C57BL/6J mice significantly declines after 24 months due to the development of comorbidities and age-related pathologies (less than 50% survival post 24 months, Yuan et al., 2009). All mice were maintained in specific pathogen free conditions with 3–5 mice/cage on corn cob bedding at Curtin University animal facilities. Prior to tumor inoculation, mice were excluded from the study if they had a palpable mass, enlarged organs (such as liver, lymph nodes, and spleen) or exhibited excessive body weight loss (>15% from age 12 months). Animals were housed in a standard light/dark cycle and sample collection performed in the morning. Experiments were performed as per the Curtin University Animal Ethics Committee (AEC) in accordance to the Australian Code of Practice for the use and care of animals for scientific purposes (AEC approval numbers: AEC_2012_21 and AEC_2016_05).

### AE17 and AE17-sOVA Murine Mesothelioma Cell Lines

AE17 is a murine malignant mesothelioma cell line generated by inoculation of asbestos fibers and was derived from orthotopic tumor deposits that emerged in elderly C57BL/6J mice. When inoculated subcutaneously (s.c.) AE17 is histologically representative of human mesothelioma (Jackaman et al., 2003). AE17 has been transfected with ovalbumin (AE17-sOVA) as a surrogate tumor marker (Jackaman et al., 2003). AE17-sOVA tumor growth and response to immunotherapy is similar to AE17. Cells were maintained in complete medium, containing RPMI 1640 (Invitrogen) supplemented with 10% fetal calf serum (FCS; ThermoScientific), 2 mM L-glutamax (Life Technologies, Victoria, VIC, Australia), 100 units/ml of penicillin, 100 μg/ml streptomycin (Life Technologies) and 0.05 mM 2-mercaptoethanol (Sigma-Aldrich) at 37°C with 5% CO_2_. For AE17sOVA this also included G418 Geneticin as a selection antibiotic (Life Technologies). Cells were collected for tumor inoculation when ≥80% confluent. Mice were inoculated s.c. with 5 × 10^5^ AE17 or AE17-sOVA cells in 100 μl PBS and body weight, body condition score and tumors monitored daily. All tumor size measurements were performed on animals using calipers and each mouse was individually tracked for tumor growth. Tumor size was determined by daily measurement of tumor width (mm) and tumor length (mm), and size calculated as width × length as mm^2^. Mice were monitored until their individual maximum tumor size reached 140 mm^2^ or weight loss exceeded >20% as per AEC conditions. For tumor-bearing mice, percentage change in body weight was calculated from the start of treatment (PBS or IL-2/anti-CD40 ± macrophage depletion).

### Macrophage Depletion and IL-2/anti-CD40 Antibody Treatment

F4/80^hi^ macrophages were depleted using anti-F4/80 antibody (similar to Tidball and Wehling-Henricks, 2007; Bedoret et al., 2009) early during tumor growth 1–9 mm^2^. Mice were treated daily, alternating between intra-peritoneal (i.p.) and intra-tumoral (i.t.) injections with 100 μg of the antibody or phosphate buffered saline (PBS; diluent control) for up to 10 days. Endotoxin levels of these antibodies are less than 0.1 EU/ml (Absolutions, Perth, WA, Australia). Proleukin IL-2 (20 μg/dose; Cetus Corporation) and anti-CD40 antibodies (40 μg/dose; FGK45, Absolutions) were used as combination immunotherapy as previously described (Jackaman et al., 2008; Jackaman and Nelson, 2012). Mice were treated intra-tumorally every 2–3 days for a total of five treatments. Mice were considered to have survived if they had complete tumor regression at 50 days following the start of treatment. We have previously confirmed that the antibody isotype does not influence tumor growth as a further control for depletion and anti-CD40 antibody (Jackaman et al., 2016).

To assess whether depletion efficiency was similar between young and elderly mice, in a separate experiment mice were sacrificed mid-way through treatment ± depletion (at day 15) when tumor size was similar between groups. Tumor samples were disaggregated into a single cell suspension by gentle dispersion between two frosted slides, followed by blocking with anti-mouse CD16/32 antibodies (Biolegend) for 15 min on ice and then staining with anti-F4/80-Alexafluor^®^ 647 for 30 min (Biolegend). Samples were then washed twice in PBS containing 2% FCS followed by acquisition on LSRFortessa™ using FACSDiva (BD Bioscience). Data were analyzed using FlowJo version 10.2 (Tree Star, Inc., Ashland, OR, United States). Macrophage depletion was effective at reducing F4/80^hi^ cells and led to a 43.9% reduction in young mice tumor samples with a 40.7% reduction in elderly mice tumor samples(Supplementary Figure S1). As a further control, to confirm that the F4/80 antibody used in flow cytometry bound a different epitope to the depleting F4/80 antibody, BM-derived macrophages were pre-incubated with depleting F4/80 antibody followed by flow cytometry staining with anti-F4/80-Alexafluor^®^ 647 antibody as described above. Pre-incubation with depleting anti-F4/80 antibody did not prevent flow cytometry staining, confirming these two antibodies bound different epitopes.

### Flow Cytometry

BM, spleen, lymph nodes, and tumors were collected into staining/collection buffer: ice-cold PBS containing 2% FCS and 2 mM EDTA (Sigma-Aldrich). Tibia and femur were flushed using a 29 g needle to isolate BM cells. Tissue samples were disaggregated into single cell suspensions with gentle dispersion between two frosted slides in staining buffer. Cells were blocked with anti-mouse CD16/32 antibodies (clone 93, Biolegend) for 15 min on ice and all subsequent steps were performed on ice in the dark. A combination of the following anti-mouse antibodies were incubated for 30 min: anti-F4/80-Alexafluor^®^ 647 (clone BM8, Biolegend), anti-CD11b-Alexafluor^®^ 488 (clone M1/70, Biolegend), anti-Ly6C-Brilliant Violet 510™ (clone HK1.4, Biolegend), anti-Ly6G-Brilliant Violet 785™ (clone 1A8, Biolegend), anti-CX3CR1-Brilliant Violet 650™ (clone SA011F11, Biolegend), and anti-CD170 (Siglec-F)-PerCP-eFluor^®^ 710 (clone 1RNM44N, eBioscience). Samples were washed in staining buffer (PBS/2%FCS/2mM EDTA), followed by PBS and incubated for 15 min with Zombie-NIR™ (Biolegend). Samples were then washed twice with PBS, followed by fixation and permeabilization with True-Nuclear Transcription Factor Buffer Set as per manufacturer’s instructions (Biolegend) for intracellular staining. Samples were incubated with anti-Ki67-PE (clone 16A8, Biolegend; diluted in True-Nuclear Transcription Factor permeabilization buffer, Biolegend), then washed twice and resuspended in staining buffer for acquisition on LSRFortessa™ using FACSDiva (BD Bioscience). Data were analyzed using FlowJo version 10.2 (Tree Star, Inc). Unstained and relevant isotype controls were included as negative controls and fluorescence minus one controls were used as gating controls.

### *In vivo* Cytotoxic T Lymphocyte (CTL) Assay

Spleen and lymph nodes from naïve C57BL/6J mice were disaggregated into a single cell suspension using two frosted glass slides, followed by pulsing with 5 μg/ml of SIINFEKL for 90 min at 37°C in RPMI/10%FCS. Uncoated control cells were also incubated for 90 min at 37°C in RPMI/10%FCS. Following incubation, the cells were washed in RPMI/10%FCS. Cells were then labeled in RPMI without FCS with either a high concentration of carboxyfluorescein succinimidyl ester (CFSE, 5 μM, SIINFEKL population) or low concentration of CFSE (0.5 μM, control cells), incubated at room temperature in the dark for 10 min. Following incubation, cells were washed twice in RPMI/10%FCS with an FCS underlay followed by two washes in PBS. For i.v. injection 1 × 10^7^ cells of each population were mixed equally in 400 μl PBS per recipient mouse. Tumor, DLNs and spleens were collected from recipient mice (young and elderly AE17-sOVA mice treated with either PBS or IL-2/anti-CD40 immunotherapy + /- macrophage depletion) 16–18 h post i.v. injection. Samples were prepared as a single cell suspension and analyzed by flow cytometry. Further controls included naïve healthy mice, AE17 tumor-bearing mice (with or without immunotherapy) and untreated recipient mice. To normalize data, percent CTL activity was expressed relative to the no tumor naïve control mice included in every experiment. Relative killing in experimental groups was calculated by determining the percentage of peptide-coated target cells relative to uncoated cells, normalized to the no tumor control mice, then multiplying by 100 to obtain a percentage value.

### Statistical Analysis

GraphPad Prism version 7 (California, CA, United States) **was** used for statistical analysis. Differences between two populations were determined using the Mann-Whitney *U*-test. A relationship between two variables was determined by Pearson’s correlation coefficient test. Results expressed as mean ± SEM. *P*-values of <0.05 were considered statistically significant.

## Results

### Mesothelioma Tumors Grow Faster in Elderly Mice

**FIGURE 1:**
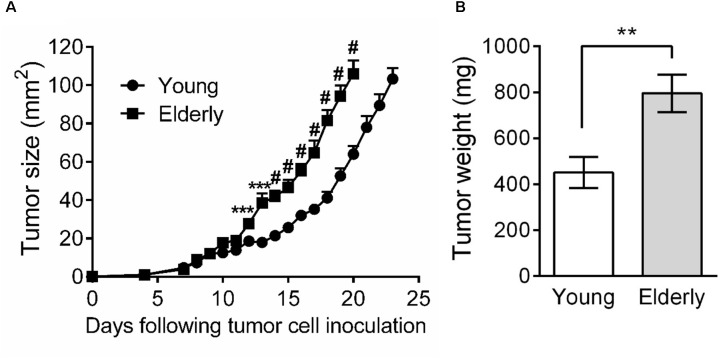
Mesothelioma tumors grow faster in elderly mice. Young (*n* = 15) and elderly (*n* = 13) C57BL/6J mice were inoculated s.c. with 5 × 10^5^ AE17 mesothelioma tumor cells and tumor growth monitored. Tumor size was determined by daily measurement of tumor width (mm) and tumor length (mm) using calipers, and size calculated as width × length as mm^2^ **(A)**. Tumor weight was recorded at endpoint (day 21–23, *n* = 7 young mice and 8 elderly mice), based on the maximum allowed size (140 mm^2^; **B**). Data is pooled from three experiments shown as mean ± SEM. ^∗∗^*p* < 0.01, ^∗∗∗^*p* < 0.005, ^#^*p* < 0.001.

As few studies have examined the effects of aging on tumor growth, including mesothelioma, we first aimed to investigate AE17 mesothelioma tumor growth in young vs. elderly mice. Tumor cells were inoculated into young and elderly C57BL/6J mice and mice monitored for tumor growth. We observed that from approximately day 10, mesothelioma tumors grew faster in elderly compared with young mice (Figure 1A). Moreover, tumor weight at the same endpoint was significantly higher in elderly mice (Figure 1B).

### Tumor-Associated Macrophages (TAMs) Increase as Tumors Get Larger

**FIGURE 2:**
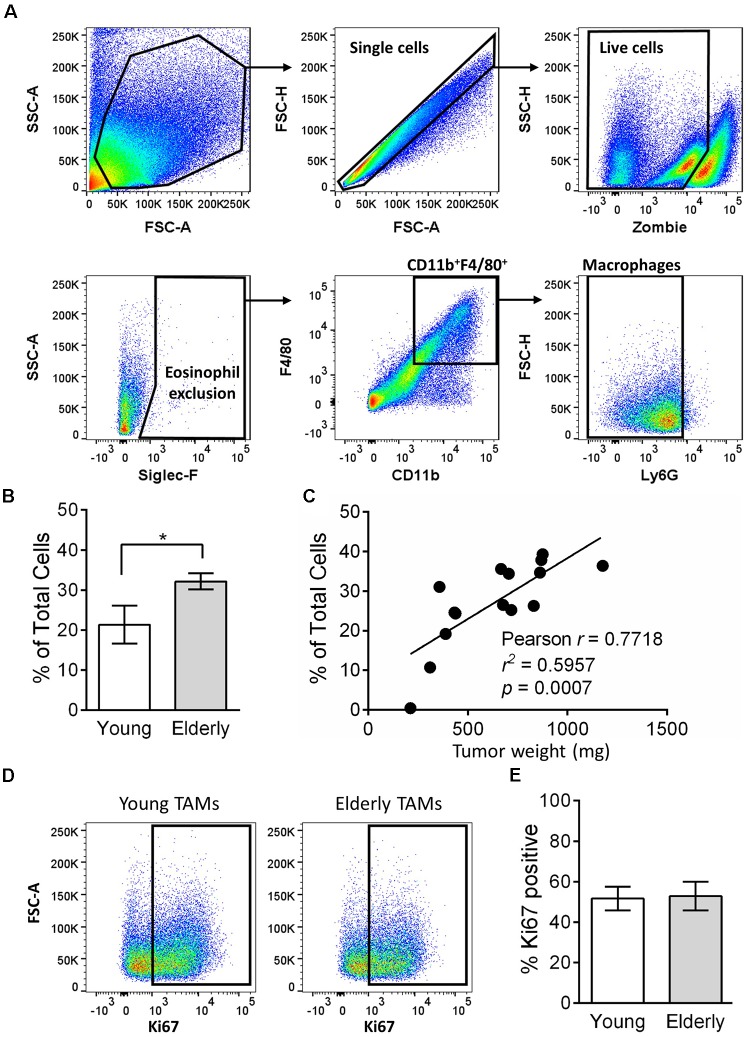
TAMs increase in tumors of elderly mice. AE17 mesothelioma tumors were collected from young (*n* = 7) and elderly C57BL/6J mice (*n* = 8) at day 21–23, based on the maximum allowed size (140 mm^2^; tumor size calculated as width × length measured using calipers). Tumor samples were dissociated into single cell suspension, stained for CD11b, F4/80, Ly6G, Siglec-F, viability dye and intracellular Ki67 and analyzed via flow cytometry. Macrophages were identified as CD11b^+^F4/80^+^Ly6G^-^Siglec-F^-^ (representative gating strategy shown in **(A)**. Macrophages are shown as % total cells **(B)** and correlated to tumor weight **(C)**. Example flow cytometry plots for Ki67 staining are shown in **(D)**, and pooled data for % proliferation (by Ki67 staining % positive) of TAMs in **(E)**. Data is pooled from three experiments shown as mean ± SEM; ^∗^*p* < 0.05.

Tumor-associated macrophages can play a key role in tumor growth, including mesothelioma (Jackaman et al., 2016), and their numbers are often associated with poor prognosis (Luo et al., 2006; Zhang et al., 2012, 2016; Fritz et al., 2014). Given the faster tumor growth in elderly mice, we next assessed the percentage of TAMs in tumors from young and elderly mice. Flow cytometry analysis (gating strategy in Figure 2A) showed that the percentage of CD11b^+^F4/80^+^Ly6G^-^Siglec-F^-^ macrophages was significantly increased in tumors of elderly compared with young mice (Figure 2B) and was associated with increasing tumor weight (Figure 2C), i.e., larger tumors have a greater percentage of macrophages. Proliferation *in situ* may account for TAMs expansion in elderly tumors, therefore we next analyzed the proliferation marker Ki67 (example flow cytometry plots in Figure 2D). In both young and elderly tumors, >50% of TAMs were proliferating, however, there were no age-related differences (Figure 2E). Furthermore, TAM proliferation was not associated with tumor size (Supplementary Figure S2). Therefore, it is possible that increased TAMs in elderly tumor-bearing mice may be due to an increased supply from other tissue sites.

### Macrophages Increase in Elderly Mice and Have Altered Proliferative Potential in Lymphoid Tissues in Response to Mesothelioma

**FIGURE 3:**
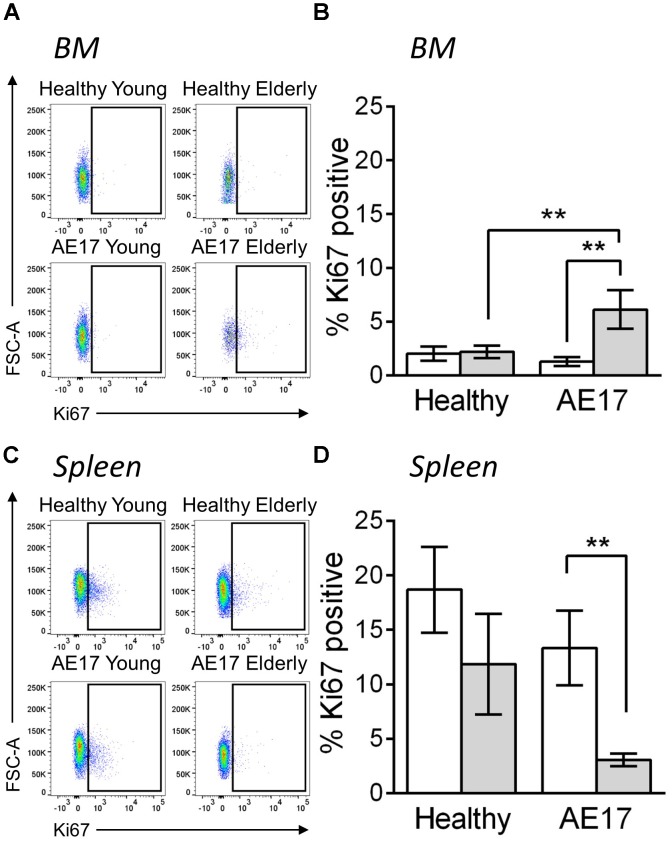
Macrophages have altered proliferative potential in lymphoid tissues in response to mesothelioma tumor. Young (*n* = 9) and elderly C57BL/6J mice (*n* = 9) were inoculated with s.c. with 5 × 10^5^ AE17 mesothelioma tumor cells and sacrificed at day 21–23, based on the maximum allowed size (140 mm^2^; tumor size calculated as width × length measured using calipers). Healthy control mice were also included (*n* = 8 for young and *n* = 9 for elderly). Spleen and bone marrow samples were dissociated into single cell suspensions, stained for CD11b, F4/80, Ly6G, Siglec-F, viability dye and intracellular Ki67 and analyzed via flow cytometry. Macrophages (CD11b^+^F4/80^+^Ly6G^-^Siglec-F^-^) were measured for proliferation via Ki67 staining (shown as % positive) in the BM (example plots shown in **A**, pooled data in **B**) and spleen (example plots shown in **C**, pooled data in **D**) of young and elderly healthy and AE17 tumor bearing mice. Data is pooled from three experiments shown as mean ± SEM. ^∗∗^*p* < 0.01.

We next examined BM and spleen to assess whether the presence of tumor influences macrophages in these tissues, particularly as these two sites can potentially contribute macrophages to the tumor (Cortez-Retamozo et al., 2012). Similar to previous studies, the proportion of macrophages (CD11b^+^F4/80^+^Ly6G^-^Siglec-F^-^) in the BM and spleen increased during healthy aging, however, this did not further increase during mesothelioma tumor growth (Wang et al., 1995; Jackaman et al., 2013; (Supplementary Figure S3). This suggests that these sites have a larger pool of macrophages in elderly mice and there may be increased potential to supply macrophages to the tumor in elderly mice. To further investigate macrophages in the BM and spleen, we measured *in vivo* proliferation via Ki67 during AE17 mesothelioma tumor growth. Whilst BM macrophage proliferation did not change in response to mesothelioma in young mice, elderly mice significantly increased proliferation compared with healthy controls and young tumor-bearing mice (example Ki67 staining in Figure 3A and pooled data in Figure 3B). In contrast, we observed decreased proliferation in splenic macrophages in elderly hosts with tumor (example Ki67 staining in Figure 3C and pooled data in Figure 3D). This suggests cross-talk occurs between the tumor and lymphoid tissues in the elderly microenvironment. This may also have the potential to further influence tumor growth in the elderly as previous studies have shown that the spleen and BM can supply macrophages to the tumor site (Cortez-Retamozo et al., 2012).

### Macrophage Depletion Improves IL-2/anti-CD40 Immunotherapy and Reduces Cachexia in Elderly Mice

**FIGURE 4:**
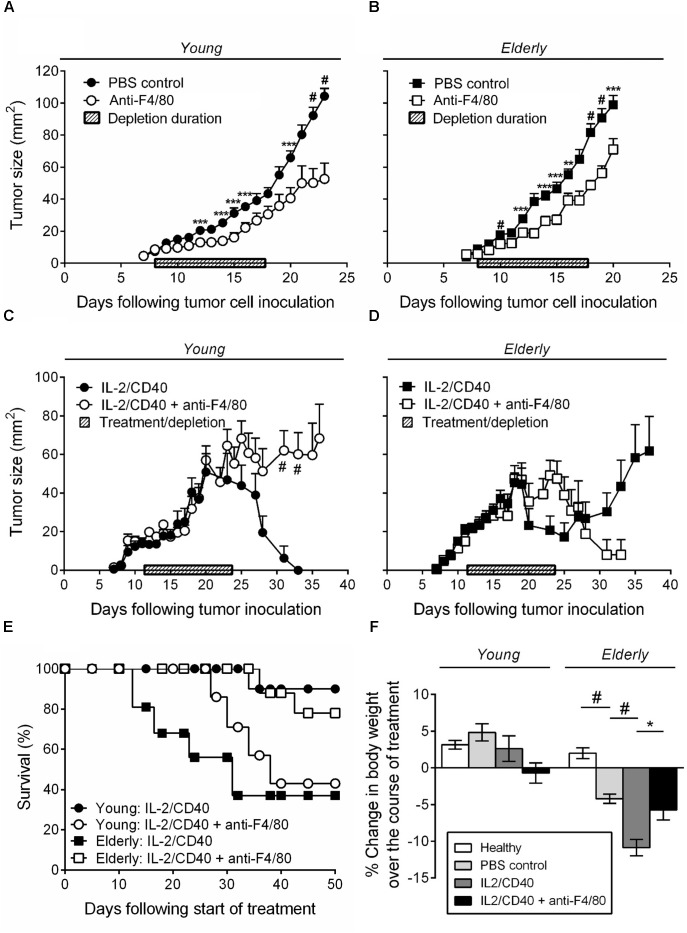
IL-2/anti-CD40 immunotherapy is less effective in elderly mice and accelerates cachexia. Young (*n* = 9/group) and elderly (*n* = 10/group) C57BL/6J mice were inoculated s.c. with 5 × 10^5^ AE17 mesothelioma tumor cells and macrophage-depleted using anti-F4/80 antibody when tumors were palpable (1–9 mm^2^; tumor size calculated as width × length measured using calipers). Mice were injected daily for 10 days, alternating between i.p. and i.t. administration (100 μg/dose in 100 μl PBS). PBS diluent or antibody isotype control was used for control mice. Pooled data from three experiments is shown for macrophage depletion, without immunotherapy, for young mice **(A)** and elderly mice **(B)** as mean ± SEM. In separate experiments, young (*n* = 10–14/group) and elderly (*n* = 9–11/group) C57BL/6J mice were inoculated s.c. with 5 × 10^5^ AE17 mesothelioma tumor cells. Macrophages were depleted using anti-F4/80 antibody 2 days prior to the start of IL-2/anti-CD40 immunotherapy. Anti-F4/80 antibody was injected daily for 10 days, alternating between i.p. and i.t. (100 μg/dose in 100 μl PBS). When tumors reached 15–21 mm^2^ (tumor size calculated as width × length measured using calipers) i.t. IL-2/anti-CD40 immunotherapy (20 μg IL-2 and 40 μg anti-CD40 Ab, 100 μl/dose in PBS) was administered every 2–3 days for five treatments. Pooled data from three experiments is shown for macrophage depletion + immunotherapy for young mice tumor growth **(C)**, elderly mice tumor growth **(D)** and survival for both **(E)** as mean ± SEM. The percentage change in body weight was calculated from the start of treatment (PBS or IL-2/anti-CD40 ± macrophage depletion) and compared to age-matched healthy controls **(F)**. Data shown as mean ± SEM. ^∗^*p* < 0.05, ^∗∗^*p* < 0.01, ^∗∗∗^*p* < 0.005, ^#^*p* < 0.001.

We have previously shown that intra-tumoral IL-2 combined with anti-CD40 antibody leads to AE17 mesothelioma tumor regression in young mice; this was associated with increased CTL activity (Jackaman et al., 2008, 2016). Furthermore, *in vitro* studies showed that elderly derived IL-2/anti-CD40-activated macrophages could restore age- and tumor-related T cell function (Jackaman et al., 2013, 2014). Therefore, we next examined whether IL-2/anti-CD40 was effective in young vs. elderly AE17 tumor-bearing mice and whether macrophages contributed to the efficacy of IL-2/anti-CD40 immunotherapy. Firstly, depletion of macrophages without immunotherapy treatment, resulted in slower tumor growth for both age groups relative to non-depleted controls (Figures 4A,B). Macrophage depletion was effective at inhibiting growth of small tumors ≤9 mm^2^ in both age groups and had no impact on tumor growth if depletion was commenced at ≥16 mm^2^ (data not shown).

Immunotherapy in young mice led to full tumor regression (Figure 4C) and 90% survival at 50 days after start of the treatment (Figure 4E). However, macrophage inhibition during IL-2/anti-CD40 immunotherapy in young mice led to tumor outgrowth (Figure 4C) and only 43% survival (Figure 4E). In contrast, immunotherapy in elderly mice was less effective in preventing tumor outgrowth (Figure 4D) and led to only 38% survival at 50 days (Figure 4E). Inhibition of macrophages in elderly mice improved the response to IL-2/anti-CD40 immunotherapy leading to a reduction in tumor growth (Figure 4D) and improved survival (78%, Figure 4E). These data show that macrophages are required for immunotherapy to be effective in young mice, yet impair responses to immunotherapy in elderly mice.

Interestingly, we also found that only elderly (and not young) tumor-bearing mice exhibited significant body weight loss (cachexia) which was severely exacerbated by the immunotherapy (Figure 4F, body weights are shown for mice examined during treatment from approximately day 12–24). This suggests that elderly mice are more susceptible to cancer cachexia, and that immunotherapy accelerates the cachexia. Furthermore, in elderly, but not young mice, macrophage inhibition prevented the IL-2/anti-CD40 immunotherapy treatment-induced cachexia (Figure 4F).

### Macrophages Play a Role in Sabotaging Anti-tumor CTLs in Elderly Tumor-Bearing Mice

**FIGURE 5:**
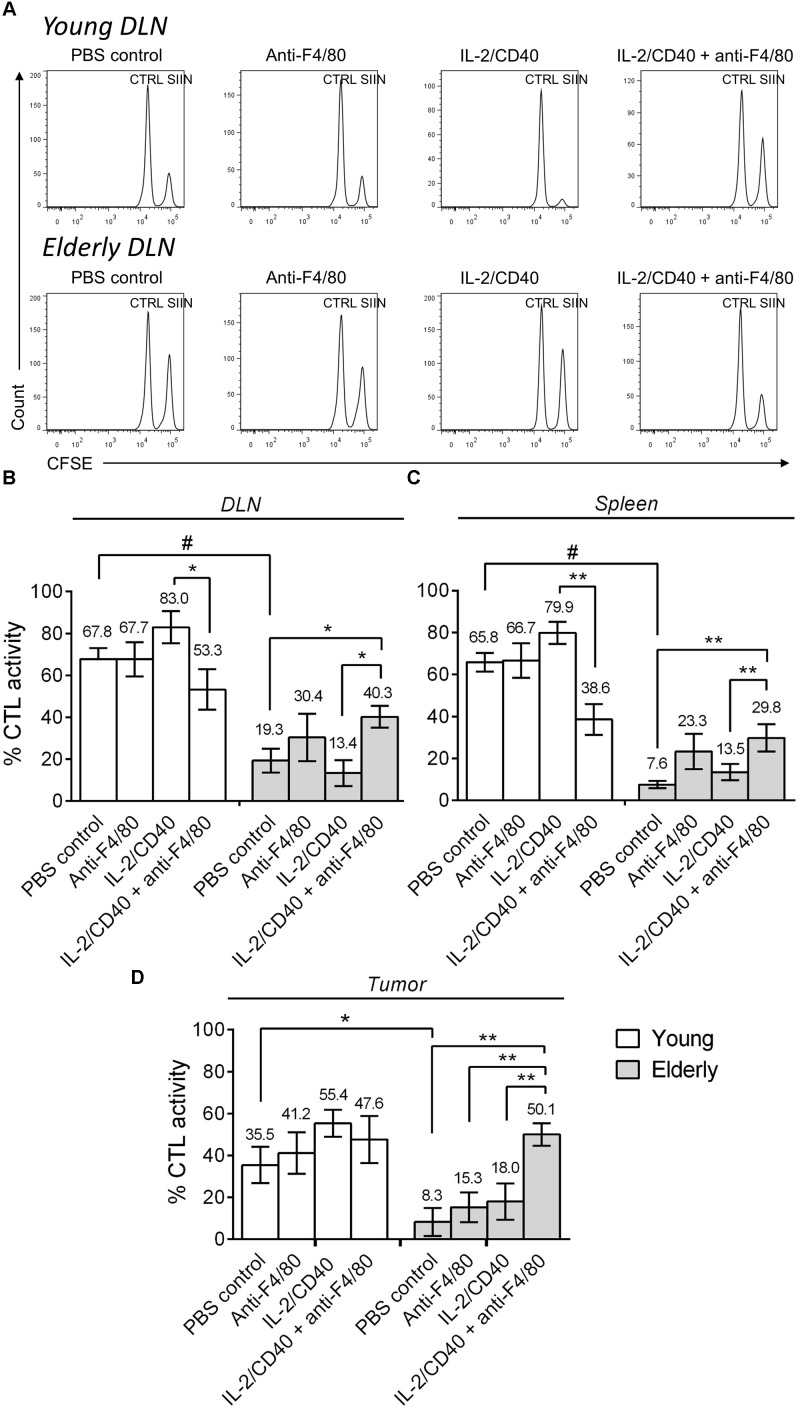
Macrophages sabotage anti-tumor CTL activity during immunotherapy in elderly mice. Young (*n* = 6–9/group) and elderly (*n* = 7–9/group) C57BL/6J mice were inoculated s.c. with 5 × 10^5^ AE17-sOVA mesothelioma tumor cells. Macrophages were depleted using anti-F4/80 antibody 2 days prior to the start of IL-2/anti-CD40 immunotherapy. Anti-F4/80 antibody was injected daily, alternating between i.p. and i.t. (100 μg/dose in 100 μl PBS). When tumors reached 15–21 mm^2^ (tumor size calculated as width × length measured using calipers) i.t. IL-2/anti-CD40 immunotherapy (20 μg IL-2 and 40 μg anti-CD40 Ab, 100 μl/dose in PBS) was administered every 2–3 days. *In vivo* CTL activity was assessed following two doses of i.t. IL-2/anti-CD40 immunotherapy when tumor size was similar between groups. Target cells pooled from LNs and spleen cells of naïve C57BL/6J mice were pulsed with SIINFEKL (SIIN) and labeled with a high concentration of CFSE. No peptide control cells (CTRL) were labeled with a low concentration of CFSE. These populations were pooled equally and injected i.v. into mice for flow cytometry analysis 16–18 h later (example DLN plots in **A**). CTL activity was calculated as a percentage, based on reduction of the SIINFEKL population relative to the no peptide control population in DLN **(B)**, spleen **(C)** and tumor **(D)**. Data is pooled from three experiments shown as mean ± SEM, with the average value above each bar graph. ^∗^*p* < 0.05, ^∗∗^*p* < 0.01, ^#^*p* < 0.001.

In order to determine whether CTL activity was altered during aging following macrophage depletion and immunotherapy, we next performed *in vivo* CTL analyses in AE17sOVA tumor-bearing mice ± IL-2/anti-CD40 immunotherapy ± macrophage depletion. In young mice, immunotherapy led to a trending increase (although non-significant) in CTL activity in the DLN, spleen and tumor (Figure 5A shows example flow cytometry plots and pooled data in Figures 5B–D). However, macrophage depletion combined with immunotherapy decreased CTL activity in the DLN and spleen of young mice. During aging, without immunotherapy, CTL activity was impaired in elderly PBS controls compared with young PBS control mice in DLN, spleen and tumor (Figure 5A example plots and pooled data in Figures 5B–D). IL-2/anti-CD40 immunotherapy alone did not significantly improve CTL activity in DLN, spleen or tumor of elderly mice. Interestingly, combining macrophage depletion with immunotherapy increased CTL activity in elderly DLN, spleen and tumor (Figures 5B–D). Overall, these data highlight that macrophages are required for CTL activity during immunotherapy in young mice, yet play a role in impairing CTL activity in elderly mice during immunotherapy.

## Discussion

Studies have shown that macrophages play a role in tumor progression (Luo et al., 2006; Zhang et al., 2012; Fritz et al., 2014; Jackaman et al., 2017) and several lines of evidence indicate these cells are dysregulated with aging (Mahbub et al., 2012; Bouchlaka et al., 2013; Jackaman et al., 2013; Oishi and Manabe, 2016; Thevaranjan et al., 2017). However, few studies have examined the role of macrophages in cancer during aging. Therefore, we aimed to examine whether macrophage dysregulation in the elderly impacted tumor progression and responses to immunotherapy. Our data showed marked differences in the growth of mesothelioma tumors and that cachexia is greatly increased by immunotherapy in elderly mice; confirming a central role for macrophages in these age-related responses. Interestingly, macrophages also played a key role in suppressing anti-tumor T cell activity during immunotherapy. These observations in mice are discussed in detail below in the context of potential implications for elderly cancer patients, efficacy of cancer immunotherapies and severity of cachexia.

Our data showed that mesothelioma tumors grew faster in elderly mice which coincided with increased TAMs. The percentage of TAMs in tumors positively correlated with tumor size rather than *in situ* TAM proliferation. Macrophages increased in the aging spleen whilst elderly BM macrophages demonstrated enhanced proliferation in response to mesothelioma. This suggests that augmented supply of BM macrophages in elderly mice may account for increased TAMs and faster tumor growth. Other studies have also shown an age-associated increase of macrophages in the spleen (Loukov et al., 2016), blood (Stranks et al., 2015), and BM (Wang et al., 1995). Furthermore, expansion of myeloid precursors is reported with aging (Dykstra et al., 2011; Pang et al., 2011; Loukov et al., 2016; Ho et al., 2017), allowing rapid production of monocyte/macrophages in response to immunological challenge (Kovtonyuk et al., 2016). This process may be linked to inflammaging, as increased circulating levels of pro-inflammatory cytokines, such as TNF-α and IL-6, can drive hematopoietic stem cells toward the myeloid lineage (Loukov et al., 2016). The increased pool of macrophage progenitors with aging may have consequences for CSF-1 producing cancers, including AE17 murine mesothelioma (Demetri et al., 1989; Fox et al., 2012). CSF-1 is a growth factor required for the proliferation, differentiation, and survival of macrophages (Pixley and Stanley, 2004). Furthermore, CSF-1 can also act as a chemoattractant for monocyte/macrophages (Shaposhnik et al., 2010). This may be important in the context of the aging microenvironment, as tumor-derived CSF-1 could circulate to stimulate macrophage population expansion in various tissues and also help to chemoattract such macrophages to the tumor, further driving macrophage-mediated tumor growth in the elderly.

Recent studies by Netea et al. (2016) in young mice and humans have described a paradigm shift in monocyte/macrophage biology in that these cells can develop a form of non-specific immunological memory or “training” (Quintin et al., 2012; Cheng et al., 2014; Saeed et al., 2014). This training results in a heightened pro-inflammatory phenotype or immune tolerance, characterized by altered cytokine production, following exposure to the same or another agent (Netea et al., 2016). Further, recent studies have described that trained immunity can occur at the level of macrophage precursors in the BM and is associated with a shift toward the myeloid lineage in the BM (Mitroulis et al., 2018), similar to the shift that occurs during aging described above (Dykstra et al., 2011; Pang et al., 2011; Loukov et al., 2016). Correspondingly, the cytokines associated with inflammaging (e.g., TNF-α, IL-6, IL-1β, MCP-1, and IL-10 (Franceschi et al., 2007) are also similar to those associated with a training phenotype. It is possible that the aging environment leads to macrophage training as suggested by others (Fulop et al., 2016; Franceschi et al., 2017) which is further enhanced by the tumor microenvironment. A similar study which also supports this hypothesis administered IL-2/anti-CD40 systemically to elderly female C57BL/6J mice (aged 16–22 months) leading to acute inflammatory-induced pathology due to macrophage-derived TNF-α production (Bouchlaka et al., 2013). Whether training is a mechanism behind macrophage dysfunction during aging remains to be determined and warrants further investigation.

Interestingly, our study showed that macrophages from the BM and spleen showed opposing proliferative capacity in response to aging and mesothelioma; with BM macrophages showing increasing proliferation and splenic macrophages decreasing proliferation. It is possible that longer period of exposure to the aging environment may account for decreased proliferative potential in terminally differentiated splenic macrophages (Stout and Suttles, 2005; Wang et al., 2013). This may be further exacerbated with cancer leading to replicative senescence in macrophages; as previously described in T cells where cancer leads to a burst of proliferation followed by senescence (Chou and Effros, 2013). Further, recent studies have suggested that macrophage origin may influence their response. BM-derived macrophages minimally contribute to tissue-resident populations during homeostasis (Ginhoux et al., 2010), instead they are recruited during inflammation or cancer development. During states of inflammation, the BM can also outsource the production of monocytes to extramedullary sites such as the spleen, leading to storage, amplification and deployment of monocytes in response to inflammatory signals or cancer (Cortez-Retamozo et al., 2012; Shand et al., 2014). Therefore, it is possible that macrophages from the spleen have been exposed for longer to the inflammatory aging microenvironment, relative to the BM, leading to a senescent phenotype; hence, reduced proliferation in aging which is further exacerbated with cancer.

We have previously shown that local IL-2/anti-CD40 immunotherapy can enhance anti-tumor T cell activity and intra-tumoral inflammation leading to resolution of mesothelioma tumors in young (aged 2–3 months) female C57BL/6J mice (Jackaman et al., 2008, 2016; Jackaman and Nelson, 2012). However, in elderly mice this was less effective and associated with a decline in T cell activity, similar to previous studies (Provinciali et al., 2000; Gravekamp et al., 2008; Castro et al., 2009). Interestingly, our data suggest that macrophages impair anti-tumor T cell responses in elderly mice during immunotherapy as macrophage depletion improved outcomes in elderly but not young mice. In contrast, macrophage depletion in young mice impaired immunotherapy and anti-tumor CTL activity. This could be via direct T cell interaction or indirectly through cytokine production and activation of dendritic cell (DC) function (Sharma et al., 2006; Gardner and Ruffell, 2016). We have previously shown that elderly derived macrophages display a heightened response to tumor-derived factors, leading to increased production of TGF-β and IL-10 (Jackaman et al., 2013). This would likely drive DCs to induce T cell tolerance in the aged tumor microenvironment (Esebanmen and Langridge, 2017). Consistent with this, our corresponding DC studies (Gardner et al., under review) show that DCs upregulate numerous checkpoint inhibitory molecules (e.g., CD73, PD-1) during aging and IL-2/anti-CD40 immunotherapy. Further studies are required to examine the cross-talk between macrophages, DCs and T cells in aging and cancer.

Cancer cachexia was evident in this study in all tumor-bearing elderly mice. Cachexia is a comorbidity condition in elderly cancer patients (Ali and Garcia, 2014) and can impair the host’s ability to tolerate anti-cancer treatments (Ross et al., 2004; Aapro et al., 2014; Kimura et al., 2015), as seen in the present study by rapid weight loss with IL-2/anti-CD40 immunotherapy for the elderly mice. Interestingly, macrophage depletion prevented treatment-induced weight loss in elderly mice suggesting a role for macrophages in cachexia. Macrophages can be a main source of pro-inflammatory mediators (Biswas and Mantovani, 2010), including TNF-α and IL-6 (Thevaranjan et al., 2017), which lead to skeletal muscle and adipose tissue catabolism, key factors in cachexia (Fearon et al., 2012; Laine et al., 2013; Batista et al., 2016). Similarly, in a previous study where IL-2/anti-CD40 was given systemically, macrophages from elderly (16–22 months) female C57BL/6J but not young mice increased production of TNF-α and IL-6 (Bouchlaka et al., 2013). Macrophage production of these cytokines is reported to increase with age in adipose tissue from elderly C57BL/6J mice (Lumeng et al., 2011) suggesting an increased risk of muscle and adipose tissue atrophy in the elderly. Therefore, in the presence of tumor-derived inflammatory signals, macrophages may play a key role in driving cancer cachexia in the elderly. Overall, our data suggests that elderly mice may be more representative of the situation for some human cancers and highly relevant to studies on the serious problem of cancer cachexia.

During healthy aging in mice and humans there is a progressive intrinsic loss of muscle mass and function termed sarcopenia (Ali and Garcia, 2014). Sarcopenia is associated with many factors including altered metabolism, denervation (Barns et al., 2014) and inflammaging (Chhetri et al., 2018). In male C57BL/6J mice sarcopenia is apparent from approximately 18 months and is pronounced by 24 months (Barns et al., 2014), whilst in humans sarcopenia increases from about 50 years of age (Sayer et al., 2013). This is taking into consideration that aging in mice occurs for approximately 9 months compared with >20 years in humans. The extent of sarcopenia varies between muscles and is more pronounced in elderly male compared with female C57BL/6J mice (White et al., 2016). Interestingly, gender is also known to influence immune responses (Klein et al., 2015); involving to some extent estrogen; (Laffont et al., 2017), although few pre-clinical studies have examined the influence of gender on anti-tumor immunity during aging. Another contributing factor to consider is the impact of the aging nervous system, since in healthy aging mice sarcopenia is also associated with changes in peripheral nerves (Krishnan et al., 2016). Pre-existing age-related neuronal changes might exacerbate the reported neurotoxic effects that have been recently reported for some immunotherapies (Stone and DeAngelis, 2016). It is not known to what extent this intrinsic propensity for sarcopenia (and additional neuronal changes) with increasing age may contribute to severity of cachexia in response to tumor load in the elderly mice. Furthermore, the influence of gender also needs to be considered in the context of age-related severity of cachexia.

In summary, these new data highlight the role of macrophages in tumor progression, particularly during aging where we observed increased proportions and altered proliferation of macrophages in the spleen and BM; two sites that can supply macrophages to the tumor site. Macrophages from elderly mice impaired anti-tumor T cell function and was associated with poorer responses to IL-2/anti-CD40 immunotherapy. Yet in young mice, macrophages were necessary for tumor regression, suggesting macrophages become dysregulated and more suppressive in elderly. Consequently, removal of dysfunctional macrophages from elderly mice improved response to treatment and prevented treatment-induced cachexia. These studies highlight the potential for targeting macrophages to improve response to anti-tumor immunotherapy in the elderly. Further animal studies are required to determine the underlying mechanisms behind macrophage dysfunction during aging, the impact on cancer immunotherapy and interventions to help reduce cancer cachexia in elderly humans.

## Author Contributions

DN and CJ conceptualized and designed the project and experiments, were responsible for the management and co-ordination of research activity, and acquired the financial support for the project. DN, CJ, FP, and MG provided the supervision for research activity planning and study execution. LD, CJ, HR-C, DD, FT, and JG performed the experiments. LD and CJ analyzed the data and drafted the manuscript. All authors contributed to writing, reviewing and editing the manuscript.

## Conflict of Interest Statement

DN acts as a non-salaried Chief Scientific Officer for Selvax. Selvax had no role in study design, data collection and analysis, decision to publish, or preparation of the manuscript. The remaining authors declare that the research was conducted in the absence of any commercial or financial relationships that could be construed as a potential conflict of interest.
