# Supplementary material for: Macrophage Depletion in Elderly Mice Improves Response to Tumor Immunotherapy, Increases Anti-tumor T Cell Activity and Reduces Treatment-Induced Cachexia
Source: Front Genet. 2018 Nov 6;9:526. doi: 10.3389/fgene.2018.00526 (PMC6232269; doi:10.3389/fgene.2018.00526)
Supplement: Supplementary file 1 [file Data_Sheet_1.PDF]

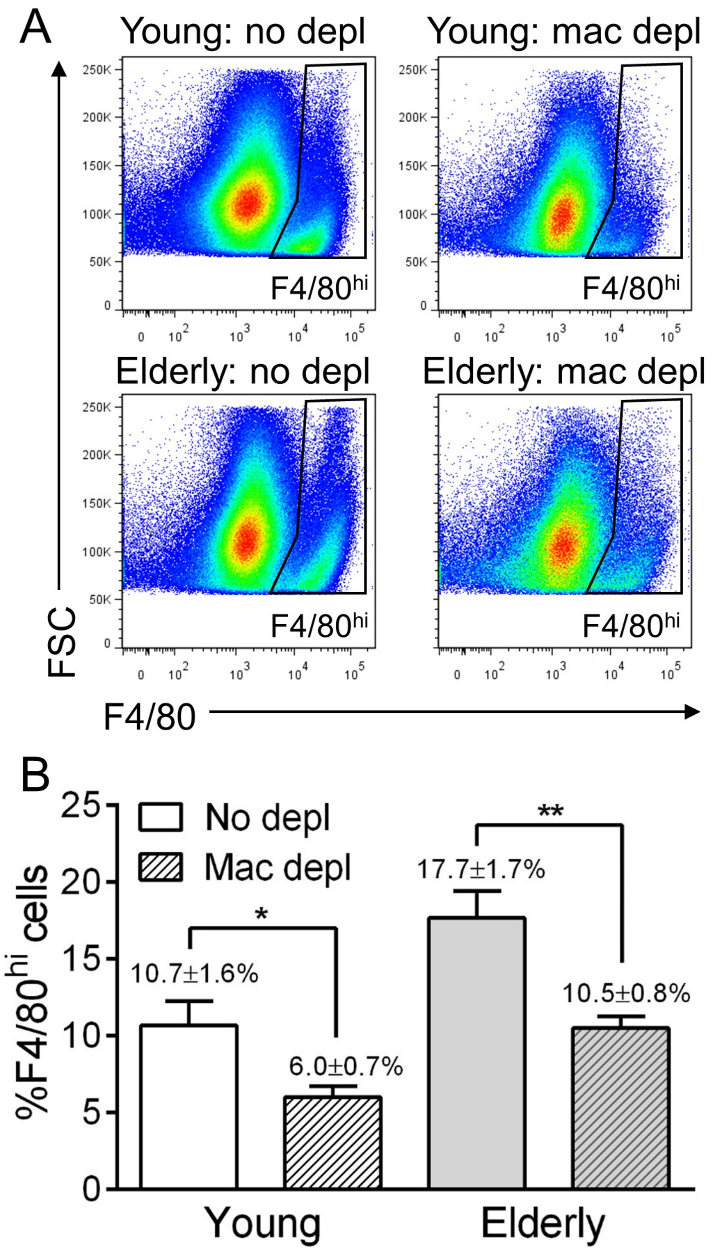

Figure S1. Macrophage depletion efficiency was similar between young and elderly mice. Mice (n = 5-7/group) were inoculated with 5 x 10^5 AE17 cells s.c. and macrophage depletion commenced at day 10 when tumors were ~9mm^2. Anti-F4/80 antibody was injected daily, alternating between i.p. and i.t. administration (100 μg/dose in 100 μl PBS). Intra-tumoral IL-2/anti-CD40 immunotherapy (20 μg IL-2 and 40 μg anti-CD40 Ab, 100 μl/dose in PBS) was administered at day 12 and day 14 and PBS diluent was used for control mice. Mice were sacrificed at day 15 when tumor size was similar between groups. Tumor samples from non-depleted and macrophage depleted groups were stained with F4/80 for analysis via flow cytometry (example plots shown in A). Macrophage depletion was only effective at reducing F4/80^{hi} cells and led to a 43.9% reduction in young mice tumor samples with a 40.7% reduction in elderly mice tumor samples (B). Data shown as mean ± SEM, * = $p < 0.05$, ** = $p < 0.01$.

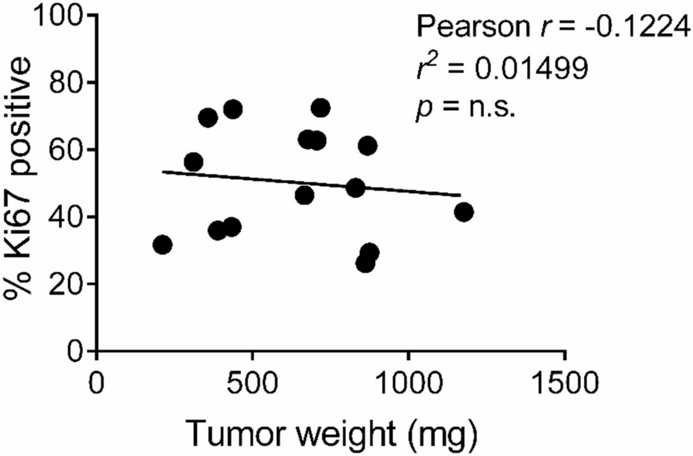

Figure S2. TAMs Ki67 proliferation is similar in both young and elderly C57BL/6J mice. Tumor samples from young (n = 7 mice) and elderly (n = 8 mice) AE17 mesothelioma tumors (day 21-23) were dissociated into single cell suspension, stained for CD11b, F4/80, Ly6G, Siglec-F, viability dye and intracellular Ki67 and analysed via flow cytometry. Tumor weight was recorded at endpoint, based on the maximum allowed size (140 mm^2). Macrophage ($CD11b^+F4/80^+Ly6G^-Siglec\text{-}F^-$ cells) proliferation (by Ki67 staining % positive) is shown correlated to tumor weight, with each dot representing an individual mouse tumor sample.

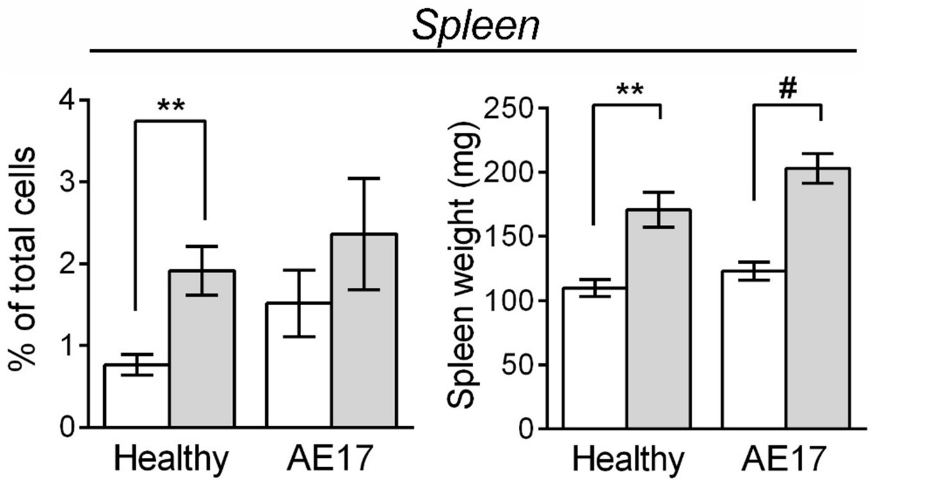

Figure S3. Spleen macrophage proportions and splenic weight increases during aging. Young (n = 9) and elderly C57BL/6J mice (n = 9) were inoculated with s.c. with 5 x 10^5 AE17 mesothelioma tumor cells and sacrificed at day 21-23, based on the maximum allowed size (140 mm^2). Healthy control mice were also included (n = 8 for young and n = 9 for elderly). Spleen samples were dissociated into single cell suspensions, stained for CD11b, F4/80, Ly6G, Siglec-F, viability dye and analysed via flow cytometry. Macrophages were identified as $CD11b^+F4/80^+Ly6G^-$ $Siglec\text{-}F^-$. Spleen weight was recorded at endpoint (day 21-23). Data is pooled from 3 experiments shown as mean ± SEM. ***p* < 0.01, # *p* < 0.001.
